# Clinical Application of Indocyanine Green Fluorescence Technology in Laparoscopic Radical Gastrectomy

**DOI:** 10.3389/fonc.2022.847341

**Published:** 2022-03-04

**Authors:** Meng Wei, Yize Liang, Limei Wang, Zhen Li, Yuanyuan Chen, Zhibo Yan, Danping Sun, Yadi Huang, Xin Zhong, Peng Liu, Wenbin Yu

**Affiliations:** ^1^ Department of Gastrointestinal Surgery, General Surgery, Qilu Hospital, Cheeloo College of Medicine, Shandong University, Jinan, China; ^2^ Department of Gastroenterology, Qilu Hospital, Cheeloo College of Medicine, Shandong University, Jinan, China; ^3^ Nursing Department, Qilu Hospital, Cheeloo College of Medicine, Shandong University, Jinan, China

**Keywords:** indocyanine green (ICG), fluorescence, gastric cancer, laparoscopic gastrectomy (LG), lymph node

## Abstract

**Background:**

This study aimed to observe the application and evaluate the feasibility and safety of indocyanine green (ICG) fluorescence technology in laparoscopic radical gastrectomy (LRG).

**Methods:**

Patients who underwent LRG & D2 lymphadenectomy at Qilu Hospital of Shandong University were included between January 2018 and August 2019. According to whether endoscopic injection of ICG was performed, patients were assigned to the ICG group (n=107) and the control group (n=88). The clinicopathologic features, retrieved lymph nodes, postoperative recovery, and follow-up data were compared between the two groups.

**Results:**

Baseline characteristics are comparable. The ICG group had a significantly larger number of lymph nodes retrieved (49.55 ± 12.72 vs. 44.44 ± 10.20, P<0.05), shorter total operation time (min) (198.22 ± 13.14 vs. 202.50 ± 9.91, P<0.05), shorter dissection time (min) (90.90 ± 5.34 vs. 93.74 ± 5.35, P<0.05) and less blood loss (ml) (27.51 ± 12.83 vs. 32.02 ± 17.99, P<0.05). The median follow-up time was 29.0 months (range 1.5-43.8 months), and there was no significant difference between the ICG group and the control group in 2-year OS (87.8% vs. 82.9%, P>0.05) or DFS (86.0% vs. 80.7%, P>0.05).

**Conclusions:**

ICG fluorescence technology in laparoscopic radical gastrectomy has advantages in LN dissection, operation time, and intraoperative blood loss. The 2-year OS and 2-year DFS rates between the two groups were comparable. In conclusion, ICG fluorescence technology is feasible and safe.

## Introduction

Gastric cancer is the fifth most frequently diagnosed cancer and the fourth leading cause of death from cancer worldwide (1). Composing complete removal of the tumor and systemic lymph node (LN) dissection, radical surgery remains the mainstay frontline treatment for resectable gastric cancer (2–4). Adequate assessment of the lymph nodes is essential for its role in the disease stage and its prognostic value (5–10), and D2 lymphadenectomy is recommended for advanced gastric cancer (2–4, 11–13).

Laparoscopic gastrectomy (LG) was first reported by Kitano (14) in 1994 and applied in the treatment of advanced gastric cancer by Goh (15) in 1997. Possessing the advantages of minimal invasion and quick postoperative recovery, LG is gradually replacing open surgery as the first choice (16–19). However, because of the lack of tactile feedback and direct observation compared with open surgery, precise tumor positioning under laparoscopy is relatively difficult, especially for patients with early gastric cancer not invading the serosa and those who need additional surgery after noncurative ESD. In addition, the complexity and vastness of the layout of blood and lymphatic vessels contribute to the difficulty and risk of effective LN dissection. Decision and evaluation making done only by the means of surgeons’ experience is extremely subjective and poses a danger of false negativity, which may cause insufficient LN dissection and poor prognosis of patients.

As a new surgery technology, dye-mediated surgical navigation (including carbon nanoparticles, indocyanine green, etc.) proved to supply surgeons with improved inspection of the complex perigastric anatomy during laparoscopic surgery. Studies have shown that carbon nanoparticle lymphatic mapping technology increases the number of LNs harvested and realizes tumor localization (20–22). Drawbacks exist, however, that once the carbon nanoparticles leaked into the abdominal cavity, the whole surgical field would be dyed black, thus interfering with the vision of the surgery field and increasing operation difficulty.

Approved by the US Food and Drug Administration (FDA) in the 1960s, ICG was applied to assess cardiac output and hepatic function in the early stage (23–26). Possessing the advantages of not interfering with the surgical field and high tissue penetration (27, 28), ICG fluorescence-guided laparoscopic surgery is therefore the subject of numerous studies (29–34). At present, the application of ICG in LRG has achieved certain success (35, 36) (37, 38). When injected into the gastric tissue around the tumor with endoscopy and exposed to a specific wavelength of near-infrared light, fluorescence emitted from ICG displays the tumor and perigastric LNs (39), making them visible and facilitating the surgery.

To further investigate the feasibility and safety of ICG fluorescence technology in LRG and provide valuable medicine evidence for clinical decision-making in radical gastric cancer resection, we conducted this retrospective study by evaluating the role of ICG fluorescence technology in surgical procedures, lymph node dissection, short-term survival, etc.

## Materials and Methods

### Patients and Study Design

Patients who underwent LRG in the Department of Gastrointestinal Surgery, Qilu Hospital of Shandong University from January 2018 to August 2019 were considered for inclusion. According to whether endoscopic injection of ICG was performed, patients were assigned to the ICG group and the control group. Endoscopic ICG injection is an invasive procedure and can only be performed with the patient’s consent. Some patients refused the endoscopic ICG injection.

The inclusion criteria were as follows: (1) Primary gastric adenocarcinoma in T1-T4a confirmed by postoperative pathology. (2) Underwent LRG + D2 lymphadenectomy.

The exclusion criteria were as follows: (1) History of previous gastrectomy, endoscopic mucosal resection, or endoscopic submucosal dissection. (2) History of other malignant diseases within the past five years. (3) History of previous neoadjuvant chemotherapy or radiotherapy. (4) Requirement of simultaneous surgery for other diseases. (5) Conversion to laparotomy.

The analyzed data were as follows: (1) Demographic data: age, sex, body mass index (BMI), American Society of Anesthesiology (ASA) physical status scores, and Eastern Cooperative Oncology ECOG performance status. (2) Perioperative outcomes: surgical procedure, operation time, blood loss, first flatus, first liquid diet, postoperative hospital stay, and postoperative complications. (3) Pathological outcomes: tumor diameter, histology, pT, and pN stage. (4) Assessment of D1 station LNs, D2 station LNS, and overall LNs. (5) Overall survival time (OS) and disease-free survival time (DFS).

The study protocol was approved by the Medical Ethics Committee of Qilu Hospital of Shandong University. All procedures were conducted under the ethical standards of the responsible committee on human experimentation (institutional and national) and with the Helsinki Declaration.

### Preoperative ICG Injection

**Figure 1:**
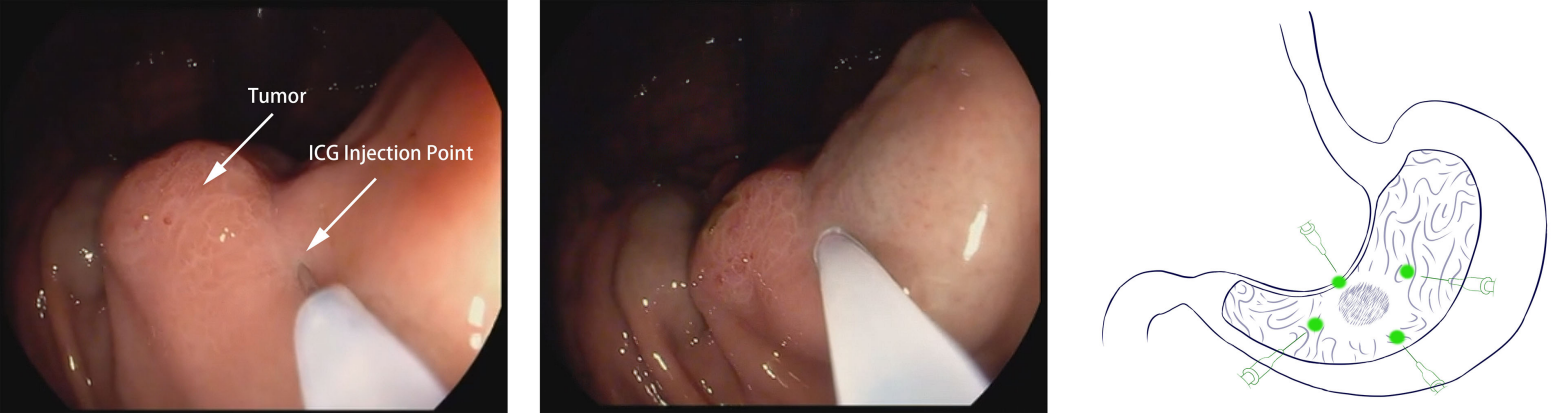
Endoscopic peritumoral ICG injection one day before surgery. A site adjacent to the tumor is selected. Slight swelling of the mucosa without ICG leakage is a sign of successful injection.

Endoscopy was performed 1 day (12-24 hours) before surgery for patients in the ICG group. Four points in the stomach (proximal, distal, and bilateral to the tumor region) were selected, and “sandwich injection methods” were used. In other words, 0.5 ml normal saline + 0.5 mL of ICG solution + 0.5 ml normal saline were injected sequentially into the submucosa layer of each point (**Figure 1**). ICG (25 mg/dose, produced by Dandong Yichuang Pharmaceutical Co., Dandong, China) was diluted with distilled water at a dose of 0.625 mg/ml. Well-trained endoscopists performed all the injections in this study to ensure accurate injection.

### Surgery Procedure

**Figure 2:**
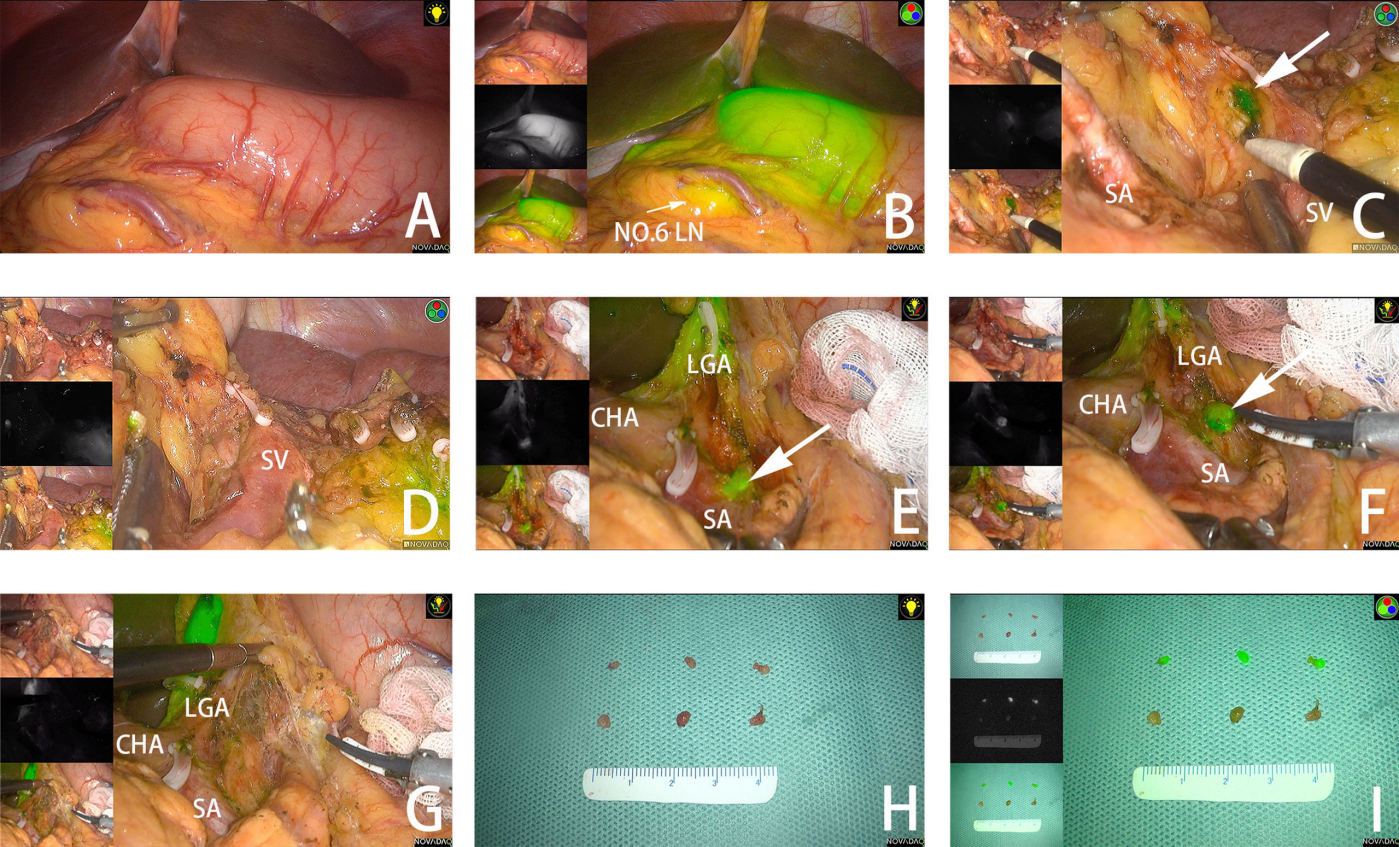
**(A, B)** The tumor is observed under white light and fluorescent mode. **(C)** The fluorescent No.10 LNs are detected under fluorescent mode. **(D)** No remnant No.10 LNs are found after dissection. **(E)** Fluorescent No. 11P LNs are adjacent to splenic vessels. **(F)** No.11P LNs are separated from blood vessels. **(G)** No remnant No. 11P LNs are found after dissection. **(H)** LNs dissected from the specimen under white light. **(I)** LNs dissected from the specimen under fluorescent mode. The arrow points to the fluorescent LN. SA, spleen artery; SV, spleen vein; CHA, common hepatic artery; LGA, left gastric artery.

In this study, a NOVADAQ fluorescence surgical system (Stryker Co., Kalamazoo, MI, USA) was applied. All patients underwent laparoscopic radical gastrectomy + D2 lymphadenectomy. During the procedure, the surgeon viewed the surgical field with frequent switching between white light view and near-infrared mode to enable accurate tumor localization (**Figures 2A, B**) and adequate lymphadenectomy at each LN station.

The gastric resection extent and lymphadenectomy were determined based on the tumor location, as stated in the Japanese guidelines (2). Total gastrectomy was performed with Roux-en-Y esophagojejunostomy, and distal gastrectomy was performed with Billroth II gastrojejunostomy + Braun anastomosis.

If fluorescent LNs were detected outside the planned dissection areas (stations 10 and 14v), excessive dissection beyond the scope of D2 lymphadenectomy was performed. (**Figures 2C, D**) In some areas with complex anatomy, such as the spleen vessels and No. 11P LNs, surgery was performed with the assistance of ICG fluorescence. (**Figures 2E–G**) After dissection of LNs in all stations, the near-infrared mode was used to assess the completeness of the lymphadenectomy and remove remnant fluorescent LNs.

### Specimen Management

A surgeon from the surgical team performed specimen management immediately after the surgery. LNs of different stations were separated from the specimen according to “the Japanese Classification of Gastric Carcinoma: 3rd English edition” (40) and separately sent to the pathology department. In addition, LNs in the ICG group were examined according to different stations and whether they were fluorescent. (**Figures 2H, I**).

### Follow Up

A minimum follow-up of 24 months was required and achieved for each patient after surgery. All enrolled patients underwent physical examination, blood testing, computed tomography, and upper gastrointestinal endoscopy regularly (41–43). Disease-free survival (DFS) time and overall survival time (DFS) were calculated.

### Statistical Analysis

The differences between the two groups were assessed using t tests, χ2 tests, or Fisher’s exact tests as appropriate. The Kaplan–Meier method and the log-rank test were used for survival analysis. All tests were 2-sided with a significance level of P < 0.05. All data were analyzed using SPSS statistical software, version 24.0 (IBM Corp., Armonk, NY, USA). The data are presented as the mean ± standard deviation for continuous variables and as a number for categorical variables.

## Results

One hundred ninety-five patients (107 patients in the ICG group and 88 patients in the control group) were retrospectively analyzed. No significant differences were observed in sex, age, BMI, ASA score, or ECOG performance status between the two groups (P > 0.05), which indicates that the baseline characteristics of the two groups were comparable. (**Table 1**)

**Table 1:** Baseline characteristics of ICG and control group.

	ICG n=107	Control n=88	P Value
Sex			0.859
Male	57 (53.3%)	48 (54.5%)	
Female	50 (46.7%)	40 (45.5%)	
Age (Years)	59.27 ± 8.99	61.53 ± 10.30	0.103
BMI (kg/m²)	24.60 ± 3.41	24.95 ± 2.65	0.424
ASA Score			0.490
I	16 (15.0%)	16 (18.2%)	
II	83 (77.5%)	62 (70.5%)	
III	8 (7.5%)	10 (11.4%)	
ECOG performance status			0.076
0	94 (87.9%)	69 (78.4%)	
1	13 (12.1%)	19 (21.6%)	

Data are shown as the mean ± standard deviation or number (%).

ICG, indocyanine green; BMI, body mass index; ASA, American Society of Anesthesiologists; ECOG, Eastern Cooperative Oncology Group.

### Clinicopathologic Characteristics

Clinicopathologic characteristics are listed in **Table 2**. No significant differences between the two groups were observed in tumor diameter, histology, pathological stage, or surgical procedure (P>0.05). Compared to the control group, the ICG group had a significantly shorter total operation time (min) (198.22 ± 13.14 vs 202.50 ± 9.91, P<0.05), shorter dissection time (min) (90.90 ± 5.34 vs 93.74 ± 5.35, P<0.05), and less blood loss (ml) (27.51 ± 12.83 vs 32.02 ± 17.99, P<0.05). There were no significant differences between the two groups in anastomosis time (min) (65.04 ± 3.89 vs 65.82 ± 4.39, P>0.05). The data were compared between the two groups, and no significant differences were observed in terms of first flatus (hours) (63.50 ± 27.345 vs 68.26 ± 28.83, P>0.05), first water intake (hours) (85.51 ± 29.03 vs 92.43 ± 28.48, P>0.05), or postoperative hospital stay (days) (9.22 ± 2.48 vs 9.26 ± 3.04, P>0.05).

**Table 2:** Perioperative outcomes of ICG and control group.

	ICG n=107	Control n=88	P Value
Tumor diameter (cm)	4.03 ± 2.48	4.09 ± 2.46	0.871
Histology			0.164
Poorly Differentiated	77 (72.0%)	52 (59.1%)	
Moderately Differentiated	21 (19.6%)	26 (29.5%)	
Well Differentiated	9 (8.4%)	10 (11.4%)	
pT stage			0.894
T1	35 (32.7%)	28 (31.8%)	
T2	18 (16.8%)	17 (19.3%)	
T3	37 (34.6%)	32 (36.4%)	
T4a	17 (15.9%)	11 (12.5%)	
pN stage			0.169
N0	50 (46.7%)	53 (60.2%)	
N1	13 (12.1%)	10 (11.4%)	
N2	11 (10.3%)	11 (12.5%)	
N3a	18 (16.8%)	7 (8.0%)	
N3b	15 (14.0%)	7 (8.0%)	
Surgical procedure			0.235
Distal gastrectomy	59 (55.1%)	41 (46.6%)	
Total gastrectomy	48 (44.9%)	47 (53.4%)	
Operation time (minute)	198.22 ± 13.14	202.50 ± 9.91	0.013
Dissection time	90.90 ± 5.34	93.74 ± 5.35	<0.001
Anastomosis time	65.04 ± 3.89	65.82 ± 4.39	0.190
Blood loss (ml)	27.51 ± 12.83	32.02 ± 17.99	0.043
First flatus (hour)	63.50 ± 27.35	68.26 ± 28.83	0.239
First water intake (hour)	85.51 ± 29.03	92.43 ± 28.48	0.096
Postoperative hospital stay (day)	9.22 ± 2.48	9.26 ± 3.04	0.931

Data are shown as the mean ± standard deviation or number (%).

Postoperative complications occurred in 15 patients (14%) in the ICG group (anastomotic bleeding in one patient, delayed gastric emptying in one, inflammatory bowel obstruction in two, pneumonia in eight, cholecystitis in two, and lymphatic leakage in one) and 12 patients (13.6%) in the control group (anastomotic leakage in one patient, delayed gastric emptying in two, pneumonia in seven, and cholecystitis in two), and there were no significant differences in the overall postoperative complication rate. (P > 0.05). According to the Clavien–Dindo classification of surgical complications, in the ICG group, 11 patients were classified as grade II or lower, 3 patients as grade IIIa, 1 patient as grade IIIb, and no patient as grade V or higher; in the control group, 7 patients were classified as grade II or lower, 4 patients as grade III a, 1 patient as grade IIIb, and no patient as grade V or higher. The distribution of severity was similar between the 2 groups. Furthermore, 1 patient in the ICG group and 1 patient in the control group experienced a repeat of surgery as a result of anastomotic leakage and bleeding. All patients with complications in both groups were discharged successfully after conservative treatment or surgical interventions. (**Table 3**)

**Table 3:** Postoperative complications of ICG and control group.

	ICG n=107	Control n=88	P Value
Postoperative complications	15 (14.0%)	12 (13.6%)	1.000
Anastomotic complication			
Bleeding	1 (0.9%)	0	1.000
Leakage	0	1 (1.1%)	1.000
Functional complication			
Delayed gastric emptying	1 (0.9%)	2 (2.3%)	1.000
Inflammatory bowel obstruction	2 (1.9%)	0	0.502
Others			
Respiratory infection	8 (7.5%)	7 (8.0%)	0.784
Cholecystitis	2 (1.9%)	2 (2.3%)	1.000
Lymphatic leakage	1 (0.9%)	0	1.000
In-hospital mortality	0	0	
Clavien–Dindo classification			0.678
I	2 (1.9%)	0	
II	9 (8.4%)	7 (8.0%)	
IIIa	3 (2.8%)	4 (4.5%)	
IIIb	1 (0.9%)	1 (1.1%)	
IV	0	0	
V	0	0	

Data are shown as number (%).

### Lymph Nodes Examination

The number of LNs harvested in the ICG group was significantly higher than that in the control group in terms of the overall LNs (49.55 ± 12.72 vs 44.44 ± 10.208, P<0.05) and the D1 station (28.54 ± 10.55 vs 24.13 ± 6.67, P<0.05), and no difference in the number of D2 station LNs was observed (21.05 ± 4.76 vs 20.38 ± 4.96, P>0.05).

The number of metastatic lymph nodes in the ICG group was significantly higher than that in the control group in terms of the overall LNs (6.45 ± 10.96 vs 3.33 ± 6.45, P<0.05) and the D1 station (5.06 ± 8.52 vs 2.40 ± 4.42, P<0.05), and no difference in the number of metastatic D2 station LNs was observed (1.39 ± 2.93 vs 0.92 ± 2.32, P>0.05). No significant differences were found in the metastatic rate of LNs in any LN classification between the two groups.

In the ICG group, there was no significant difference in the positive rate of LNs between fluorescent and nonfluorescent LNs (**Table 4**).

**Table 4:** Number of retrieved lymph nodes in the ICG and control groups & Positive rate in the ICG group of fluorescent and nonfluorescent LNs.

	ICG n=107	Control n=88	P Value
Overall LNs			
Total LNs	49.55 ± 12.72	44.44 ± 10.20	0.002
Positive LNs	6.45 ± 10.96	3.33 ± 6.45	0.014
Positive rate	9.96 ± 17.83%	12.20 ± 22.38%	0.438
D1 Station LNs			
Total LNs	28.54 ± 10.55	24.13 ± 6.67	<0.001
Positive LNs	5.06 ± 8.52	2.40 ± 4.42	0.006
Positive rate	11.49 ± 18.84%	13.71 ± 21.74%	0.446
D2 Station LNs			
Total LNs	21.05 ± 4.76	20.38 ± 4.96	0.337
Positive LNs	1.39 ± 2.93	0.92 ± 2.32	0.221
Positive rate	5.07 ± 11.63%	5.55 ± 11.49%	0.774
	**fluorescent LNs**	**nonfluorescent LNs**	**P Value**
Positive rate	17.27 ± 27.58%	9.80 ± 21.39%	0.370

Data are shown as the mean ± standard deviation or number (%).

### Two Years Follow-Up

All patients were followed up, and data were collected: the median follow-up for all patients was 29.0 months (range 1.5-43.8 months). At the time of the last follow-up on August 31, 2021, 162 patients (83%) were alive without recurrence (90 in the ICG group and 72 in the control group), and 6 patients (3%) were alive with recurrence (4 in the ICG group and 2 in the control group). Twenty-seven of 195 patients (14%) had died; among them, 10 patients (5%) in the ICG group had recurrence at the time of death (2 patients with locoregional recurrence, 4 patients with local and distant recurrence, and 4 patients with distant recurrence) and 12 patients (6%) in the control group (2 patients with locoregional recurrence, 7 patients with local and distant recurrence, and 3 patients with distant recurrence), and 5 patients (3%) died due to other causes in the two groups (**Table 5**).

**Table 5:** Patients’ status at last follow-up.

	ICG n=107	Control n=88	P Value
2-year OS	87.8%	82.9%	0.304
2-year DFS	86.0%	80.7%	0.471
2-year OS in T1	93.5%	92.0%	0.814
2-year DFS in T1	94.3%	92.9%	0.806
2-year OS in T2	94.4%	85.6%	0.472
2-year DFS in T2	94.4%	82.4%	0.275
2-year OS in T3	94.2%	85.0%	0.212
2-year DFS in T3	89.2%	87.5%	0.918
2-year OS in T4	52.9%	32.9%	0.191
2-year DFS in T4	52.9%	27.3%	0.175
Alive	94 (87.9%)	74 (84.1%)	
Alive without recurrence	90 (84.1%)	72 (81.8%)	
Alive with recurrence	4 (3.7%)	2 (2.3%)	
Death	13 (12.1%)	14 (15.9%)	
Death with other causes	3 (2.8%)	2 (2.3%)	
Death with recurrence	10 (9.3%)	12 (13.6%)	
Locoregional	2 (1.9%)	2 (2.3%)	
Local and distant	4 (3.7%)	7 (8.0%)	
Distant	4 (3.7%)	3 (3.4%)	

Data are shown as number (%).

**Figure 3:**
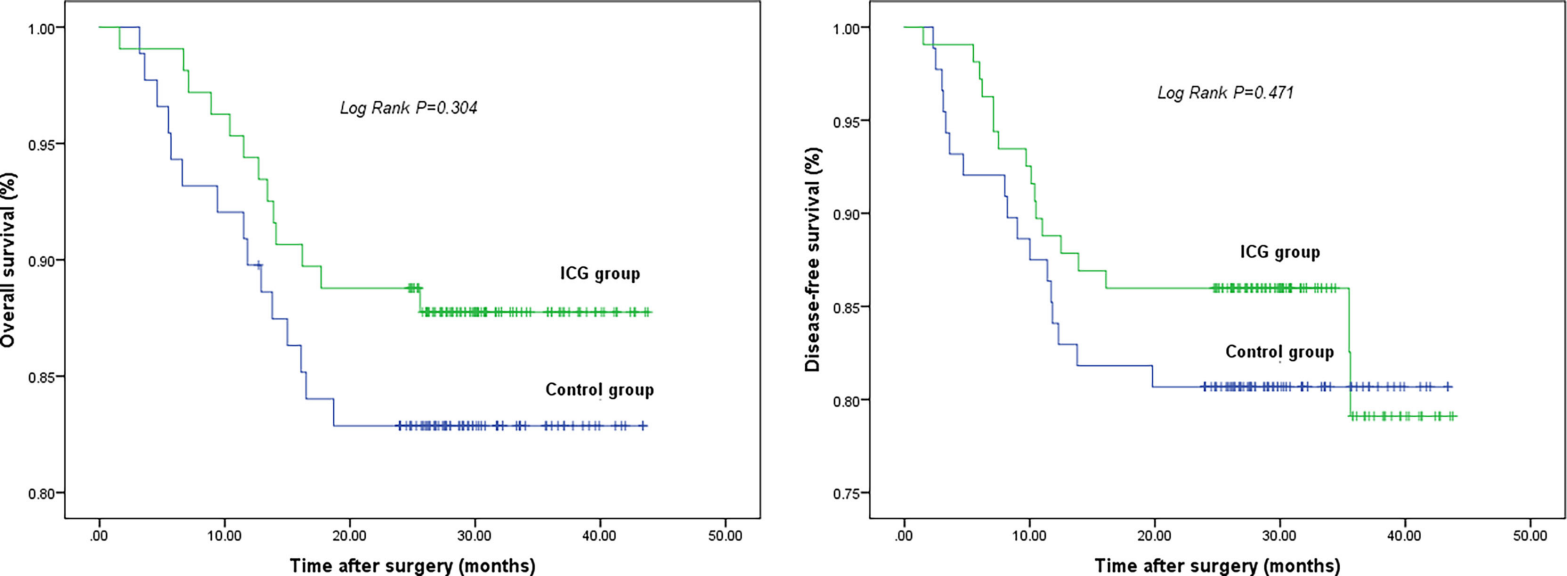
2-year overall survival (left) and 2-year disease-free survival (right). There is no difference in 2-year OS and 2-year DFS between two groups.

The long-term survival did not show differences between the ICG and control groups: the 2-year OS was 87.8% in the ICG group and 82.9% in the control group (log-rank p = 0.304). The 2-year DFS was 86.0% in the ICG group and 80.7% in the control group (log-rank p = 0.471). (**Figure 3**)

## Discussion

Recently, with the widespread application of laparoscopic surgery for patients with gastric cancer, ICG fluorescence-guided LRG has attracted much attention as a novel navigation technology. To evaluate the feasibility and safety of ICG in LRG, this study was conducted and indicated that compared with conventional LRG, ICG-guided LRG has the advantages of more lymph nodes dissected, less blood loss, and shorter operation time.

As a crucial step in gastric cancer surgery, adequate resection and assessment of LNs have been shown to be linked to disease staging, regional disease control, and long-term survival (6, 7, 44). Interestingly, fluorescence observation based on the absorption characteristics of ICG has been reported to make it possible to distinguish LNs containing ICG particles from surrounding tissue (45, 46), improving the chance of complete dissection. Kwon et al. (47) reported that ICG fluorescence-guided lymphography offered increased lymph node retrieval compared with conventional laparoscopic surgery. Chen et al. (48) suggested that more lymph nodes could be harvested during ICG-guided LRG with decreased lymph node noncompliance. Similar results are reported in this study; likewise, some new and interesting findings are yielded.

For example, the splenic artery may turn, twist, and become tortuous after originating from the celiac trunk, leading to the obscuration and difficulty of 11P LN dissection. However, possessing the property of strong tissue penetration, ICG fluorescence may highlight lymph nodes and enable the retrieval of a higher number of lymph nodes. In addition, because of the different diffusion and uptake rates of ICG in different tissues, perigastric blood vessels and associated lymphatic vessels can be accurately distinguished, making it possible to remove more lymph nodes adjacent to the blood vessel. Especially in the splenic hilum area, the relatively narrow operating space, the complexity of the vascular anatomy and the fragile texture of the spleen make ICG an indispensable tool to improve the safety and accuracy of surgery.

Since 1997, the Union for International Cancer Control (UICC) and American Joint Commission for Cancer (AJCC) have adopted the N stage of the tumor, node, metastasis (TNM) classification based on the number of metastatic lymph nodes (49). In our study, it seems that there were sufficient lymph nodes in the control group for positive lymph node status determination and more lymph nodes in the ICG group were unnecessary, but we should not ignore the fact that lymph node micrometastasis could not be exactly evaluated by routine H&E examination and is well associated with poor outcome in patients (50–54). Huang et al. (55) demonstrated that the number of dissected LNs is the only factor affecting negative lymph node counts. In other words, more lymph nodes dissected increases the number of positive lymph nodes and so-called negative nodes that may harbor micrometastases, thus leading to a better prognosis (56–58).

Although our study found that more overall LNs contributed to more positive LNs in the ICG group, we should take it into account that ICG is not a targeting marker for tumor cells (59) and is unable to trace positive LNs specifically. We also conducted a small sample (35 patients) study examining fluorescent and nonfluorescent LNs respectively in the ICG group, and no significant difference was found in the metastatic rate confirmed by pathology. Moreover, it is not uncommon to find discrepancies in which some obviously enlarged LNs are proven to be pathologically metastatic, but they are not fluorescent (60). This is considered to be partly because the lymphatic vessels were obstructed by a massive cancer embolus, the ICG cannot flow into these LNs (61). Therefore, ICG fluorescence technology can only be used to assist lymph node dissection, instead of being relied on to determine whether the lymph node is metastatic, which is consistent with the report of Cianchi et al. (62)

Another key step in curative gastric cancer surgery is the complete removal of the primary tumor with sufficient negative margins. Indeed, positive margins are associated with significantly worse survival (63–65). In fact, ICG fluorescence can improve the lack of visual inspection and palpation in traditional laparoscopic surgery and prove effective in the identification of tumors in our study. The surgeon can observe the tumor with frequent switching between white light view and near-infrared mode after endoscopic ICG injection around the tumor. As a consequence, it is possible to reduce the operation time and surgical invasion. In addition, the characteristics of ICG in distinguishing lymph nodes and surrounding tissues also enable prompt and accurate intraoperative decisions to speed up the surgical process and reduce the risk of blood vessel injury and bleeding.

It is reported that intraoperative blood loss and transfusion are associated with a higher risk of morbidity and mortality, although further investigation is needed (66–69). Yasuda et al. reported that not only the volume of blood loss, but the operation time is associated with morbidity after gastrectomy (70). In addition, cardiopulmonary adverse effects of general anesthesia and dioxide pneumoperitoneum are significant in laparoscopic surgery (71, 72). In this study, the ICG group was shown to provide the advantages of decreased intraoperative blood loss and shorter surgery time than the control group. The routine use of ICG fluorescence could potentially reduce the perioperative complications caused by blood loss and prolong dioxide pneumoperitoneum during LRG. In addition, the morbidity rates were 14.0% in the ICG group and 13.6% in the control group (P>0.05), which were similar to the rates reported in previous studies (73, 74). No intraoperative events or delayed complications during their hospital stay related to ICG were observed.

After a 2-year follow-up, there was no significant difference in long-term survival in each stage between the ICG group and the control group. This may be explained by the fact of the shorter follow up period of the present study. We also found that ICG-guided LRG obviously improved OS and DFS, because the number of harvested LNs in the ICG group was significantly larger and adequate numbers of LNs dissected in the standard lymphadenectomy region were necessary for accurate disease staging and avoiding LN micrometastasis, thus having a good impact on the prognosis of patients (75–77).

Currently, two ICG injection methods are used: preoperative endoscopic submucosal injection and intraoperative subserous injection. During the study, we found that the former is superior (61) since intraoperative injection may increase surgery risk caused by prolonged operation time and pneumoperitoneum time. In addition, the location of the tumor cannot be indicated accurately under laparoscopy if ICG was not injected around the tumor (78). There was also not sufficient time for ICG to diffuse from injection sites into the D2 station LNs. Of note, the concentration of ICG solution should not be too high; otherwise, the excessively strong fluorescence intensity may obstruct the observation of tissues. However, unlike carbon nanoparticles, low-dose of ICG is not visible in white light mode, so high concentrations or leakage of ICG do not interfere with the surgeon’s vision.

There are some limitations to this study. First, compared with the control group, patients in ICG group had to bear more costs for indocyanine green and endoscopic injection. Second, there was no significant difference in long-term survival between the two groups due to the relatively short follow-up time, so a longer follow-up is necessary. Third, ICG is not a targeting tracer for tumor cells, so efforts to develop more targeted dyes are required. Fourth, this was not a strictly randomized controlled study, but patients almost randomly accept endoscopic ICG injections. The study was conducted at Qilu hospital of Shandong university that performs more than 1,000 gastrectomies for advanced gastric cancer each year. Considering the large number of patients, relatively few inpatient beds and the cost of hospitalization, the number of days in hospital before surgery was strictly controlled. In our hospital, gastroenterologists are also endoscopists, who are mainly responsible for the treatment of patients in addition to endoscopy. Inpatients waiting for surgery can receive endoscopic injection only after completing all preoperative examinations and making a successful appointment with an endoscopist. Those who do not meet these requirements cannot receive ICG injection.

In summary, a large randomized, multicenter trial is warranted to further evaluate the feasibility and safety of indocyanine green fluorescence technology in LRG for gastric cancer.

This study indicates that, with a shorter operation time, less blood loss, and no complications attributable to ICG, ICG fluorescence technology can guide surgeons to rapidly locate tumors and harvest more lymph nodes than conventional LRG. In addition, the two-year OS and DFS are comparable between two groups. In conclusion, ICG fluorescence technology in laparoscopic radical gastrectomy is safe and valuable.

## Authors Contributions

WY is the corresponding author. MW and YZ are joint first authors. WY contributed to the study concept and design. WY, ZB and MW conducted the laparoscopic radical gastrectomy. LM and ZL conducted the endoscopy. MW and YZ wrote the manuscript. MW, YZ, LM, ZL, YY, ZB, DP, YD, XZ and PL conducted the data collection and analysis. WY revised and edited the manuscript. WY and YY are the guarantors of this study. All authors contributed to the article and approved the submitted version.

## Data Availability Statement

The raw data supporting the conclusions of this article will be made available by the authors, without undue reservation.

## Ethics Statement

The studies involving human participants were reviewed and approved by the Medical Ethics Committee of Qilu Hospital of Shandong University. The patients/participants provided their written informed consent to participate in this study.

## Funding

This work was supported by the Natural Science Foundation of Shandong Province, China (Grant/Award Number: ZR2019LZL006) and the Horizontal Project of Shandong University (Clinical Study of Intraoperative NIR Application—Clinical Study of ICG-labeled Fluorescent Laparoscopic Technique in Total Laparoscopic Radical Gastrectomy; Grant/Award Number: 6010119083).

## Conflict of Interest

The authors declare that the research was conducted in the absence of any commercial or financial relationships that could be construed as a potential conflict of interest.

## Publisher’s Note

All claims expressed in this article are solely those of the authors and do not necessarily represent those of their affiliated organizations, or those of the publisher, the editors and the reviewers. Any product that may be evaluated in this article, or claim that may be made by its manufacturer, is not guaranteed or endorsed by the publisher.

## Abbreviations

ICG, indocyanine green; LRG, laparoscopic radical gastrectomy.

## References

[1] Sung H Ferlay J Siegel RL Laversanne M Soerjomataram I Jemal A. Global Cancer Statistics 2020: GLOBOCAN Estimates of Incidence and Mortality Worldwide for 36 Cancers in 185 Countries. CA Cancer J Clin (2021) 71(3):209–49. doi: 10.3322/caac.21660 33538338

[2] Japanese Gastric Cancer A. Japanese Gastric Cancer Treatment Guidelines 2018 (5th Edition). Gastric Cancer (2021) 24(1):1–21. doi: 10.1007/s10120-020-01042-y 32060757 PMC7790804

[3] Smyth EC Nilsson M Grabsch HI van Grieken NC Lordick F. Gastric Cancer. Lancet (2020) 396(10251):635–48. doi: 10.1016/S0140-6736(20)31288-5 32861308

[4] Guideline Committee of the Korean Gastric Cancer Association DWG Review P. Korean Practice Guideline for Gastric Cancer 2018: An Evidence-Based, Multi-Disciplinary Approach. J Gastric Cancer (2019) 19(1):1–48. doi: 10.5230/jgc.2019.19.e8 30944757 PMC6441770

[5] Wu CW Hsiung CA Lo SS Hsieh MC Chen JH Li AF. Nodal Dissection for Patients With Gastric Cancer: A Randomised Controlled Trial. Lancet Oncol (2006) 7(4):309–15. doi: 10.1016/S1470-2045(06)70623-4 16574546

[6] Songun I Putter H Kranenbarg EM Sasako M van de Velde CJ. Surgical Treatment of Gastric Cancer: 15-Year Follow-Up Results of the Randomised Nationwide Dutch D1D2 Trial. Lancet Oncol (2010) 11(5):439–49. doi: 10.1016/S1470-2045(10)70070-X 20409751

[7] Amin MB Greene FL Edge SB Compton CC Gershenwald JE Brookland RK. The Eighth Edition AJCC Cancer Staging Manual: Continuing to Build a Bridge From a Population-Based to a More "Personalized" Approach to Cancer Staging. CA Cancer J Clin (2017) 67(2):93–9. doi: 10.3322/caac.21388 28094848

[8] Wu CW Hsieh MC Lo SS Tsay SH Lui WY P'Eng FK. Relation of Number of Positive Lymph Nodes to the Prognosis of Patients With Primary Gastric Adenocarcinoma. Gut (1996) 38(4):525–7. doi: 10.1136/gut.38.4.525 PMC1383108 8707081

[9] Siewert JR Bottcher K Stein HJ Roder JD. Relevant Prognostic Factors in Gastric Cancer: Ten-Year Results of the German Gastric Cancer Study. Ann Surg (1998) 228(4):449–61. doi: 10.1097/00000658-199810000-00002 PMC1191515 9790335

[10] Smith DD Schwarz RR Schwarz RE. Impact of Total Lymph Node Count on Staging and Survival After Gastrectomy for Gastric Cancer: Data From a Large US-Population Database. J Clin Oncol (2005) 23(28):7114–24. doi: 10.1200/JCO.2005.14.621 16192595

[11] Smyth EC Verheij M Allum W Cunningham D Cervantes A Arnold D. Gastric Cancer: ESMO Clinical Practice Guidelines for Diagnosis, Treatment and Follow-Up. Ann Oncol (2016) 27(suppl 5):v38–49. doi: 10.1093/annonc/mdw350 27664260

[12] Hu Y Huang C Sun Y Su X Cao H Hu J. Morbidity and Mortality of Laparoscopic Versus Open D2 Distal Gastrectomy for Advanced Gastric Cancer: A Randomized Controlled Trial. J Clin Oncol (2016) 34(12):1350–7. doi: 10.1200/JCO.2015.63.7215 26903580

[13] Degiuli M Reddavid R Tomatis M Ponti A Morino M Sasako M. D2 Dissection Improves Disease-Specific Survival in Advanced Gastric Cancer Patients: 15-Year Follow-Up Results of the Italian Gastric Cancer Study Group D1 Versus D2 Randomised Controlled Trial. Eur J Cancer (2021) 150:10–22. doi: 10.1016/j.ejca.2021.03.031 33887514

[14] Kitano S Iso Y Moriyama M Sugimachi K. Laparoscopy-Assisted Billroth I Gastrectomy. Surg Laparosc Endosc (1994) 4(2):146–8. 8180768

[15] Goh PM Khan AZ So JB Lomanto D Cheah WK Muthiah R. Early Experience With Laparoscopic Radical Gastrectomy for Advanced Gastric Cancer. Surg Laparosc Endosc Percutan Tech (2001) 11(2):83–7. doi: 10.1097/00129689-200104000-00003 11330389

[16] Lee HJ Hyung WJ Yang HK Han SU Park YK An JY. Short-Term Outcomes of a Multicenter Randomized Controlled Trial Comparing Laparoscopic Distal Gastrectomy With D2 Lymphadenectomy to Open Distal Gastrectomy for Locally Advanced Gastric Cancer (KLASS-02-RCT). Ann Surg (2019) 270(6):983–91. doi: 10.1097/SLA.0000000000003217 30829698

[17] Katai H Mizusawa J Katayama H Morita S Yamada T Bando E. Survival Outcomes After Laparoscopy-Assisted Distal Gastrectomy Versus Open Distal Gastrectomy With Nodal Dissection for Clinical Stage IA or IB Gastric Cancer (JCOG0912): A Multicentre, Non-Inferiority, Phase 3 Randomised Controlled Trial. Lancet Gastroenterol Hepatol (2020) 5(2):142–51. doi: 10.1016/S2468-1253(19)30332-2 31757656

[18] Yu J Huang C Sun Y Su X Cao H Hu J. Effect of Laparoscopic vs Open Distal Gastrectomy on 3-Year Disease-Free Survival in Patients With Locally Advanced Gastric Cancer: The CLASS-01 Randomized Clinical Trial. JAMA (2019) 321(20):1983–92. doi: 10.1001/jama.2019.5359 PMC6547120 31135850

[19] Chen QY Zhong Q Liu ZY Huang XB Que SJ Zheng WZ. Advances in Laparoscopic Surgery for the Treatment of Advanced Gastric Cancer in China. Eur J Surg Oncol (2020) 46(10 Pt B):e7–e13. doi: 10.1016/j.ejso.2020.07.015 32709375

[20] Yan J Zheng XL Liu ZYZ Yu J Deng ZW Xue FQ. A Multicenter Study of Using Carbon Nanoparticles to Show Sentinel Lymph Nodes in Early Gastric Cancer. Surg Endosc (2016) 30(4):1294–300. doi: 10.1007/s00464-015-4358-8 26150223

[21] Feng Y Yang K Sun HH Liu YP Zhang D Zhao Y. Value of Preoperative Gastroscopic Carbon Nanoparticles Labeling in Patients Undergoing Laparoscopic Radical Gastric Cancer Surgery. Surg Oncol (2021) 38:101628. doi: 10.1016/j.suronc.2021.101628 34174770

[22] Tian Y Lin Y Guo H Hu Y Li Y Fan L. Safety and Efficacy of Carbon Nanoparticle Suspension Injection and Indocyanine Green Tracer-Guided Lymph Node Dissection During Robotic Distal Gastrectomy in Patients With Gastric Cancer. Surg Endosc (2021). doi: 10.1007/s00464-021-08630-8 PMC9001219 34254184

[23] Alford R Simpson HM Duberman J Hill GC Ogawa M Regino C. Toxicity of Organic Fluorophores Used in Molecular Imaging: Literature Review. Mol Imaging (2009) 8(6):341–54. doi: 10.2310/7290.2009.00031 20003892

[24] Cherrick GR Stein SW Leevy CM Davidson CS. Indocyanine Green: Observations on its Physical Properties, Plasma Decay, and Hepatic Extraction. J Clin Invest (1960) 39:592–600. doi: 10.1172/JCI104072 13809697 PMC293343

[25] Desmettre T Devoisselle JM Mordon S. Fluorescence Properties and Metabolic Features of Indocyanine Green (ICG) as Related to Angiography. Surv Ophthalmol (2000) 45(1):15–27. doi: 10.1016/S0039-6257(00)00123-5 10946079

[26] Reinhart MB Huntington CR Blair LJ Heniford BT Augenstein VA. Indocyanine Green: Historical Context, Current Applications, and Future Considerations. Surg Innov (2016) 23(2):166–75. doi: 10.1177/1553350615604053 26359355

[27] Schaafsma BE Mieog JS Hutteman M van der Vorst JR Kuppen PJ Lowik CW. The Clinical Use of Indocyanine Green as a Near-Infrared Fluorescent Contrast Agent for Image-Guided Oncologic Surgery. J Surg Oncol (2011) 104(3):323–32. doi: 10.1002/jso.21943 PMC3144993 21495033

[28] Vahrmeijer AL Hutteman M van der Vorst JR van de Velde CJ Frangioni JV. Image-Guided Cancer Surgery Using Near-Infrared Fluorescence. Nat Rev Clin Oncol (2013) 10(9):507–18. doi: 10.1038/nrclinonc.2013.123 PMC3755013 23881033

[29] Boni L David G Mangano A Dionigi G Rausei S Spampatti S. Clinical Applications of Indocyanine Green (ICG) Enhanced Fluorescence in Laparoscopic Surgery. Surg Endosc (2015) 29(7):2046–55. doi: 10.1007/s00464-014-3895-x PMC4471386 25303914

[30] Boni L David G Dionigi G Rausei S Cassinotti E Fingerhut A. Indocyanine Green-Enhanced Fluorescence to Assess Bowel Perfusion During Laparoscopic Colorectal Resection. Surg Endosc (2016) 30(7):2736–42. doi: 10.1007/s00464-015-4540-z PMC4912584 26487209

[31] Wang X Teh CSC Ishizawa T Aoki T Cavallucci D Lee SY. Consensus Guidelines for the Use of Fluorescence Imaging in Hepatobiliary Surgery. Ann Surg (2021) 274(1):97–106. doi: 10.1097/SLA.0000000000004718 33351457

[32] Aoki T Koizumi T Mansour DA Fujimori A Kusano T Matsuda K. Ultrasound-Guided Preoperative Positive Percutaneous Indocyanine Green Fluorescence Staining for Laparoscopic Anatomical Liver Resection. J Am Coll Surg (2020) 230(3):e7–12. doi: 10.1016/j.jamcollsurg.2019.11.004 31756381

[33] Herrera-Almario G Patane M Sarkaria I Strong VE. Initial Report of Near-Infrared Fluorescence Imaging as an Intraoperative Adjunct for Lymph Node Harvesting During Robot-Assisted Laparoscopic Gastrectomy. J Surg Oncol (2016) 113(7):768–70. doi: 10.1002/jso.24226 PMC4964277 27021142

[34] Yoshida M Kubota K Kuroda J Ohta K Nakamura T Saito J. Indocyanine Green Injection for Detecting Sentinel Nodes Using Color Fluorescence Camera in the Laparoscopy-Assisted Gastrectomy. J Gastroenterol Hepatol (2012) 27(Suppl 3):29–33. doi: 10.1111/j.1440-1746.2012.07067.x 22486868

[35] Ushimaru Y Omori T Fujiwara Y Yanagimoto Y Sugimura K Yamamoto K. The Feasibility and Safety of Preoperative Fluorescence Marking With Indocyanine Green (ICG) in Laparoscopic Gastrectomy for Gastric Cancer. J Gastrointest Surg (2019) 23(3):468–76. doi: 10.1007/s11605-018-3900-0 30084063

[36] Ikoma N Badgwell BD Mansfield P. Fluorescent-Image Guidance in Robotic Subtotal Gastrectomy. Ann Surg Oncol (2020) 27(13):5322. doi: 10.1245/s10434-020-08523-5 32382891

[37] Shoji Y Kumagai K Kamiya S Ida S Nunobe S Ohashi M. Prospective Feasibility Study for Single-Tracer Sentinel Node Mapping by ICG (Indocyanine Green) Fluorescence and OSNA (One-Step Nucleic Acid Amplification) Assay in Laparoscopic Gastric Cancer Surgery. Gastric Cancer (2019) 22(4):873–80. doi: 10.1007/s10120-018-00919-3 30603913

[38] Kim TH Kong SH Park JH Son YG Huh YJ Suh YS. Assessment of the Completeness of Lymph Node Dissection Using Near-Infrared Imaging With Indocyanine Green in Laparoscopic Gastrectomy for Gastric Cancer. J Gastric Cancer (2018) 18(2):161–71. doi: 10.5230/jgc.2018.18.e19 PMC6026716 29984066

[39] Luo S Zhang E Su Y Cheng T Shi C. A Review of NIR Dyes in Cancer Targeting and Imaging. Biomaterials (2011) 32(29):7127–38. doi: 10.1016/j.biomaterials.2011.06.024 21724249

[40] Japanese Gastric Cancer A. Japanese Classification of Gastric Carcinoma: 3rd English Edition. Gastric Cancer (2011) 14(2):101–12. doi: 10.1007/s10120-011-0041-5 21573743

[41] Hur H Song KY Park CH Jeon HM. Follow-Up Strategy After Curative Resection of Gastric Cancer: A Nationwide Survey in Korea. Ann Surg Oncol (2010) 17(1):54–64. doi: 10.1245/s10434-009-0676-1 19777193

[42] Whiting J Sano T Saka M Fukagawa T Katai H Sasako M. Follow-Up of Gastric Cancer: A Review. Gastric Cancer (2006) 9(2):74–81. doi: 10.1007/s10120-006-0360-0 16767361

[43] Zanotti D Baiocchi GL Coniglio A Mohammadi B Ministrini S Mughal M. Follow-Up After Surgery for Gastric Cancer: How to do it. Updates Surg (2018) 70(2):293–9. doi: 10.1007/s13304-018-0524-6 29582358

[44] Maehara Y Kakeji Y Koga T Emi Y Baba H Akazawa K. Therapeutic Value of Lymph Node Dissection and the Clinical Outcome for Patients With Gastric Cancer. Surgery (2002) 131(1 Suppl):S85–91. doi: 10.1067/msy.2002.119309 11821792

[45] Kim DW Jeong B Shin IH Kang U Lee Y Park YS. Sentinel Node Navigation Surgery Using Near-Infrared Indocyanine Green Fluorescence in Early Gastric Cancer. Surg Endosc (2019) 33(4):1235–43. doi: 10.1007/s00464-018-6401-z 30167947

[46] Roh CK Choi S Seo WJ Cho M Son T Kim HI. Indocyanine Green Fluorescence Lymphography During Gastrectomy After Initial Endoscopic Submucosal Dissection for Early Gastric Cancer. Br J Surg (2020) 107(6):712–9. doi: 10.1002/bjs.11438 32031248

[47] Kwon IG Son T Kim HI Hyung WJ. Fluorescent Lymphography-Guided Lymphadenectomy During Robotic Radical Gastrectomy for Gastric Cancer. JAMA Surg (2019) 154(2):150–8. doi: 10.1001/jamasurg.2018.4267 PMC6439673 30427990

[48] Chen QY Xie JW Zhong Q Wang JB Lin JX Lu J. Safety and Efficacy of Indocyanine Green Tracer-Guided Lymph Node Dissection During Laparoscopic Radical Gastrectomy in Patients With Gastric Cancer: A Randomized Clinical Trial. JAMA Surg (2020) 155(4):300–11. doi: 10.1001/jamasurg.2019.6033 32101269

[49] Sano T Coit DG Kim HH Roviello F Kassab P Wittekind C. Proposal of a New Stage Grouping of Gastric Cancer for TNM Classification: International Gastric Cancer Association Staging Project. Gastric Cancer (2017) 20(2):217–25. doi: 10.1007/s10120-016-0601-9 PMC4992472 26897166

[50] Kim JJ Song KY Hur H Hur JI Park SM Park CH. Lymph Node Micrometastasis in Node Negative Early Gastric Cancer. Eur J Surg Oncol (2009) 35(4):409–14. doi: 10.1016/j.ejso.2008.05.004 18573635

[51] Yasuda K Adachi Y Shiraishi N Inomata M Takeuchi H Kitano S. Prognostic Effect of Lymph Node Micrometastasis in Patients With Histologically Node-Negative Gastric Cancer. Ann Surg Oncol (2002) 9(8):771–4. doi: 10.1007/BF02574499 12374660

[52] Lee E Chae Y Kim I Choi J Yeom B Leong AS. Prognostic Relevance of Immunohistochemically Detected Lymph Node Micrometastasis in Patients With Gastric Carcinoma. Cancer (2002) 94(11):2867–73. doi: 10.1002/cncr.10562 12115374

[53] Wu ZY Li JH Zhan WH He YL Wan J. Effect of Lymph Node Micrometastases on Prognosis of Gastric Carcinoma. World J Gastroenterol (2007) 13(30):4122–5. doi: 10.3748/wjg.v13.i30.4122 PMC4205317 17696234

[54] Zeng YJ Zhang CD Dai DQ. Impact of Lymph Node Micrometastasis on Gastric Carcinoma Prognosis: A Meta-Analysis. World J Gastroenterol (2015) 21(5):1628–35. doi: 10.3748/wjg.v21.i5.1628 PMC4316106 25663783

[55] Huang CM Lin JX Zheng CH Li P Xie JW Lin BJ. Effect of Negative Lymph Node Count on Survival for Gastric Cancer After Curative Distal Gastrectomy. Eur J Surg Oncol (2011) 37(6):481–7. doi: 10.1016/j.ejso.2011.01.012 21371852

[56] Harrison LE Karpeh MS Brennan MF. Extended Lymphadenectomy Is Associated With a Survival Benefit for Node-Negative Gastric Cancer. J Gastrointest Surg (1998) 2(2):126–31. doi: 10.1016/S1091-255X(98)80002-4 9834407

[57] Saito H Fukumoto Y Osaki T Fukuda K Tatebe S Tsujitani S. Prognostic Significance of Level and Number of Lymph Node Metastases in Patients With Gastric Cancer. Ann Surg Oncol (2007) 14(5):1688–93. doi: 10.1245/s10434-006-9314-3 17245613

[58] Deng JY Liang H Sun D Pan Y Zhang RP Wang BG. Outcome in Relation to Numbers of Nodes Harvested in Lymph Node-Positive Gastric Cancer. Eur J Surg Oncol (2009) 35(8):814–9. doi: 10.1016/j.ejso.2008.11.007 19111430

[59] Egloff-Juras C Bezdetnaya L Dolivet G Lassalle HP. NIR Fluorescence-Guided Tumor Surgery: New Strategies for the Use of Indocyanine Green. Int J Nanomedicine (2019) 14:7823–38. doi: 10.2147/IJN.S207486 PMC6768149 31576126

[60] Miyashiro I Hiratsuka M Kishi K Takachi K Yano M Takenaka A. Intraoperative Diagnosis Using Sentinel Node Biopsy With Indocyanine Green Dye in Gastric Cancer Surgery: An Institutional Trial by Experienced Surgeons. Ann Surg Oncol (2013) 20(2):542–6. doi: 10.1245/s10434-012-2608-8 22941164

[61] Tajima Y Yamazaki K Masuda Y Kato M Yasuda D Aoki T. Sentinel Node Mapping Guided by Indocyanine Green Fluorescence Imaging in Gastric Cancer. Ann Surg (2009) 249(1):58–62. doi: 10.1097/SLA.0b013e3181927267 19106676

[62] Cianchi F Indennitate G Paoli B Ortolani M Lami G Manetti N. The Clinical Value of Fluorescent Lymphography With Indocyanine Green During Robotic Surgery for Gastric Cancer: A Matched Cohort Study. J Gastrointest Surg (2020) 24(10):2197–203. doi: 10.1007/s11605-019-04382-y 31485904

[63] Songun I Bonenkamp JJ Hermans J van Krieken JH van de Velde CJ. Prognostic Value of Resection-Line Involvement in Patients Undergoing Curative Resections for Gastric Cancer. Eur J Cancer (1996) 32A(3):433–7. doi: 10.1016/0959-8049(95)00591-9 8814687

[64] Cho BC Jeung HC Choi HJ Rha SY Hyung WJ Cheong JH. Prognostic Impact of Resection Margin Involvement After Extended (D2/D3) Gastrectomy for Advanced Gastric Cancer: A 15-Year Experience at a Single Institute. J Surg Oncol (2007) 95(6):461–8. doi: 10.1002/jso.20731 17192913

[65] Morgagni P Garcea D Marrelli D de Manzoni G Natalini G Kurihara H. Does Resection Line Involvement Affect Prognosis in Early Gastric Cancer Patients? An Italian Multicentric Study. World J Surg (2006) 30(4):585–9. doi: 10.1007/s00268-005-7975-x 16547613

[66] Glance LG Dick AW Mukamel DB Fleming FJ Zollo RA Wissler R. Association Between Intraoperative Blood Transfusion and Mortality and Morbidity in Patients Undergoing Noncardiac Surgery. Anesthesiology (2011) 114(2):283–92. doi: 10.1097/ALN.0b013e3182054d06 21239971

[67] Al-Refaie WB Parsons HM Markin A Abrams J Habermann EB. Blood Transfusion and Cancer Surgery Outcomes: A Continued Reason for Concern. Surgery (2012) 152(3):344–54. doi: 10.1016/j.surg.2012.06.008 22938895

[68] Spence RK Carson JA Poses R McCoy S Pello M Alexander J. Elective Surgery Without Transfusion: Influence of Preoperative Hemoglobin Level and Blood Loss on Mortality. Am J Surg (1990) 159(3):320–4. doi: 10.1016/S0002-9610(05)81227-9 2305940

[69] Wu WC Smith TS Henderson WG Eaton CB Poses RM Uttley G. Operative Blood Loss, Blood Transfusion, and 30-Day Mortality in Older Patients After Major Noncardiac Surgery. Ann Surg (2010) 252(1):11–7. doi: 10.1097/SLA.0b013e3181e3e43f 20505504

[70] Yasuda K Shiraishi N Adachi Y Inomata M Sato K Kitano S. Risk Factors for Complications Following Resection of Large Gastric Cancer. Br J Surg (2001) 88(6):873–7. doi: 10.1046/j.0007-1323.2001.01782.x 11412261

[71] Galizia G Prizio G Lieto E Castellano P Pelosio L Imperatore V. Hemodynamic and Pulmonary Changes During Open, Carbon Dioxide Pneumoperitoneum and Abdominal Wall-Lifting Cholecystectomy. A Prospective, Randomized Study. Surg Endosc (2001) 15(5):477–83. doi: 10.1007/s004640000343 11353965

[72] Sharma KC Brandstetter RD Brensilver JM Jung LD. Cardiopulmonary Physiology and Pathophysiology as a Consequence of Laparoscopic Surgery. Chest (1996) 110(3):810–5. doi: 10.1378/chest.110.3.810 8797429

[73] Kim W Song KY Lee HJ Han SU Hyung WJ Cho GS. The Impact of Comorbidity on Surgical Outcomes in Laparoscopy-Assisted Distal Gastrectomy: A Retrospective Analysis of Multicenter Results. Ann Surg (2008) 248(5):793–9. doi: 10.1097/SLA.0b013e3181887516 18948806

[74] Kim HH Han SU Kim MC Hyung WJ Kim W Lee HJ. Long-Term Results of Laparoscopic Gastrectomy for Gastric Cancer: A Large-Scale Case-Control and Case-Matched Korean Multicenter Study. J Clin Oncol (2014) 32(7):627–33. doi: 10.1200/JCO.2013.48.8551 24470012

[75] Macalindong SS Kim KH Nam BH Ryu KW Kubo N Kim JY. Effect of Total Number of Harvested Lymph Nodes on Survival Outcomes After Curative Resection for Gastric Adenocarcinoma: Findings From an Eastern High-Volume Gastric Cancer Center. BMC Cancer (2018) 18(1):73. doi: 10.1186/s12885-017-3872-6 29329569 PMC5766983

[76] Pan S Wang P Xing Y Li K Wang Z Xu H. Retrieved Lymph Nodes From Different Anatomic Groups in Gastric Cancer: A Proposed Optimal Number, Comparison With Other Nodal Classification Strategies and its Impact on Prognosis. Cancer Commun (Lond) (2019) 39(1):49. doi: 10.1186/s40880-019-0394-4 31519217 PMC6743096

[77] Giuliani A Caporale A Corona M Di Bari M Demoro M Ricciardulli T. Lymphadenectomy in Gastric Cancer: Influence on Prognosis of Lymph Node Count. J Exp Clin Cancer Res (2004) 23(2):215–24. 15354405

[78] Kitagawa Y Fujii H Kumai K Kubota T Otani Y Saikawa Y. Recent Advances in Sentinel Node Navigation for Gastric Cancer: A Paradigm Shift of Surgical Management. J Surg Oncol (2005) 90(3):147–51; discussion 51-2. doi: 10.1002/jso.20220 15895450

